# Multicellular *In vitro* Models of Cardiac Arrhythmias: Focus on Atrial Fibrillation

**DOI:** 10.3389/fcvm.2020.00043

**Published:** 2020-03-31

**Authors:** Pim R. R. van Gorp, Serge A. Trines, Daniël A. Pijnappels, Antoine A. F. de Vries

**Affiliations:** Laboratory of Experimental Cardiology, Department of Cardiology, Leiden University Medical Center, Leiden, Netherlands

**Keywords:** *in vitro* model, disease modeling, arrhythmia research, atrial fibrillation, primary cardiomyocyte, (induced) pluripotent stem cell-derived cardiomyocyte, (conditionally) immortalized cardiomyocyte

## Abstract

Atrial fibrillation (AF) is the most common cardiac arrhythmia in clinical practice with a large socioeconomic impact due to its associated morbidity, mortality, reduction in quality of life and health care costs. Currently, antiarrhythmic drug therapy is the first line of treatment for most symptomatic AF patients, despite its limited efficacy, the risk of inducing potentially life-threating ventricular tachyarrhythmias as well as other side effects. Alternative, in-hospital treatment modalities consisting of electrical cardioversion and invasive catheter ablation improve patients' symptoms, but often have to be repeated and are still associated with serious complications and only suitable for specific subgroups of AF patients. The development and progression of AF generally results from the interplay of multiple disease pathways and is accompanied by structural and functional (e.g., electrical) tissue remodeling. Rational development of novel treatment modalities for AF, with its many different etiologies, requires a comprehensive insight into the complex pathophysiological mechanisms. Monolayers of atrial cells represent a simplified surrogate of atrial tissue well-suited to investigate atrial arrhythmia mechanisms, since they can easily be used in a standardized, systematic and controllable manner to study the role of specific pathways and processes in the genesis, perpetuation and termination of atrial arrhythmias. In this review, we provide an overview of the currently available two- and three-dimensional multicellular *in vitro* systems for investigating the initiation, maintenance and termination of atrial arrhythmias and AF. This encompasses cultures of primary (animal-derived) atrial cardiomyocytes (CMs), pluripotent stem cell-derived atrial-like CMs and (conditionally) immortalized atrial CMs. The strengths and weaknesses of each of these model systems for studying atrial arrhythmias will be discussed as well as their implications for future studies.

## Introduction

Atrial fibrillation (AF) is a rapidly growing global health problem mainly due to aging of the human population and adherence to unhealthy lifestyles. AF is associated with significant morbidity and mortality predominately resulting from embolic stroke and heart failure (1, 2). In 2010, an estimated 33.5 million people were suffering from AF worldwide with tremendous socioeconomic costs. The AF prevalence is likely underestimated as a large proportion of individuals with no or transient symptoms remain undiagnosed. Current AF therapies rely on modulation of the heart's electrical function through drugs, electrical cardioversions and invasive ablation procedures. Antiarrhythmic pharmacotherapy (i.e., pharmacological rhythm control) represents the initial treatment for most symptomatic AF patients, but is associated with side effects including negative inotropy and potentially fatal ventricular proarrhythmia (3, 4). Pharmacological rate control is usually indicated for asymptomatic patients and for older patients (with few co-morbidities) as well as in case of serious adverse effects of antiarrhythmic drugs (5, 6). Alternative treatment modalities consist of electrical cardioversions and invasive catheter ablation procedures, which must be performed in the hospital and often need to be repeated, i.e., the immediate and 1-year success rate of electrical shock therapy is 70 and 42%, respectively (7), and the 1-year AF recurrence rate following catheter ablation is 45–89% depending on the patient characteristics (8, 9). Moreover, ablation procedures inevitably lead to the loss of some contractile tissue and have a 4–5% risk of major complications (9). Nonetheless, AF ablation has been shown to decrease arrhythmia recurrences in patients with paroxysmal AF (pAF). It, however, has been less successful in patients with (longstanding) persistent AF (perAF) (10–12).

The differences in treatment outcome between distinct AF patient populations (pAF *versus* perAF) is a reflection of the heterogeneous and progressive nature of this disorder due to the involvement and interplay of multiple disease pathways. This is supported by the various cardiac conditions (e.g., congestive heart failure, structural heart disease), genetic variants and other factors (e.g., hypertension, diabetes mellitus, obesity, obstructive sleep apnea and alcohol consumption) that are known (i) to be associated with an increased risk of developing AF and (ii) to contribute to disease progression (13–15). The main pathophysiological processes involved in the development of AF are induced by mechanical and oxidative stress, inflammation and/or aberrant neuroendocrine signaling and consist of tissue fibrosis (structural remodeling) as well as changes in (i) the expression, cellular distribution and activity of ion channels, exchangers and pumps and of gap junctions (electrical remodeling), (ii) ATP production (metabolic remodeling) and (iii) (para)sympathetic signaling (autonomous remodeling) (16–18). This creates a substrate, in which the presence of focal ectopic activity (trigger) may initiate reentrant electrical activity, which comes with the formation of freely rotating and anchored reentrant waves (19–21). In the nineties, the pulmonary veins (PVs) have been shown to be a major source of focal ectopic activity and to play an important role in the genesis of AF (4, 22). Nevertheless, there's an ongoing debate about (i) the precise mechanism(s) involved in the initiation, maintenance and perpetuation of AF and (ii) the role of focal ectopic drivers from the PVs in each of these processes (23, 24). Therefore, a better understanding of the pathophysiological processes and arrhythmia mechanisms underlying AF will help to improve its management (25).

Several experimental models (*in silico, in vitro, ex vivo, in vivo*) are currently being used to study atrial arrhythmia mechanisms, all of which have their own advantages and limitations (20). To generate relevant models for studying the mechanisms involved in atrial arrhythmias, it's essential to be able to recapitulate the patterns of action potential (AP) propagation occurring in the human heart during both sinus rhythm and arrhythmia. Apart from being able to mimic anatomical and functional reentry as well as high-frequency focal activity, in order to dissect the role of specific disease pathways or arrhythmia mechanisms, it's important for the model to be applicable in a standardized, systemic and controllable manner and to allow pharmaceutical and genetic interventions. *In vivo* models are most physiological but are necessarily restricted to animals and generally display considerable biological variation, which complicates the interpretation of results. It is, however, a desirable feature in arrhythmia studies focusing on inter-individual differences. Biological and especially technical variation also have to be taken into account working with *ex vivo* models (whole organs or tissue pieces), which typically permit measurements for only a short period of time due to their gradual deterioration. Moreover, results obtained *ex vivo* using human atrial appendages should be interpreted with caution as their properties may differ from those of atrial free walls. Additionally, these appendages seem to have a limited role as source of reentry and arrhythmia in the majority of AF patients (26–29). *In silico* models are most useful for rapid testing of various conditions (in different situations) in a cheap and reproducible manner. However, as present-day mathematical models are far from being able to capture the intricacies and subtleties of complex biological systems like the atria, *in silico* findings require validation in biological systems. *In vitro* models consisting of two-dimensional (2D) monolayers or sheets of atrial myocytes (AMs), obtained by seeding of enzymatically derived cell suspensions, form functional syncytia of excitable and contractile cells, which can be readily exposed to drugs and genetically modified (30). This allows evaluation of therapeutic interventions in a controlled environment mimicking many of the features of intact atria. 2D cultures of AMs may also be subjected to other manipulations (e.g., electrical/optical pacing, ischemia-reperfusion injury, oxidative stress, dysglycemia, cyclic stretch) making them highly attractive model systems to bridge the gap between single cells and intact tissue and between computer simulations and *ex/in vivo* experiments. Due to the limited and fluctuating supply of human atrial tissue and the difficulty to maintain primary AMs of human adults in culture, several alternative 2D multicellular *in vitro* models have been developed, encompassing primary (newborn) animal-derived AMs, (pluripotent) stem-cell derived atrial(-like) cardiomyocytes (CMs) of animal and human origin as well as (conditionally) immortalized rodent AMs. Although these models lack the three-dimensional (3D) properties and complex architecture of atria with blood vessels and other cell types besides AMs (e.g., cardiac fibroblasts and autonomic neurons), they are highly suited as systems to study mechanisms and processes involved in the initiation, propagation and termination of AF.

In this review, we provide a comprehensive overview of the currently available 2D multicellular *in vitro* models that can be used to investigate the electrophysiological mechanisms involved in the initiation, continuation and termination of AF as well as the influence of various pathophysiological mechanisms on these processes. We will also briefly discuss recently developed 3D multicellular *in vitro* models of atrial arrhythmias.

## *In vitro* Multicellular Systems for AF Modeling

**Figure 1:**
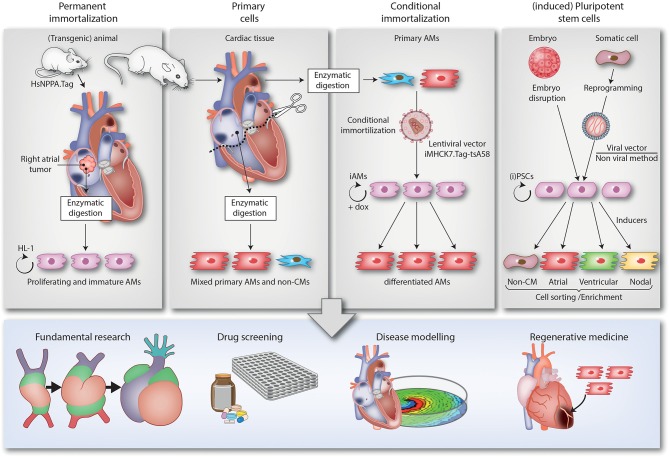
Overview of the different 2D multicellular *in vitro* models of AF. The figure is divided in 4 top panels describing the different techniques to generate 2D multicellular *in vitro* atrial arrhythmia models and a bottom panel depicting the various applications of these models (i.e., fundamental research, drug screening, disease modeling and regenerative medicine). The 1st panel from the left describes the generation of the HL-1 cell line. This cell line was derived from a transgenic mice in which expression of SV40 Tag is driven by the atrium-specific human NPPA promoter (HsNPPA-Tag). This resulted in mice carrying right atrial tumors, which were isolated and enzymatically dissociated yielding cells that could be kept in culture as the HL-1 cell line. This cell line can proliferate while retaining an immature AM phenotype. The 2nd panel from the left shows how AMs and non-CMs (e.g., fibroblasts) can be derived from atrial tissue samples of animals (e.g., neonatal rats) by enzymatic digestion and cultured *in vitro*. The resulting mixed atrial cell preparations also served as starting material for the conditional immortalization procedure depicted in the 3rd panel from the left, which led to the generation of iAM cell lines including iAM-1. The conditional immortalization was achieved by transducing primary AMs with a repressor-based lentiviral Tet-on system (represented by the lentiviral particle), in which the muscle-specific promoter MHCK7 drives the expression of a temperature-sensitive mutant of SV40 Tag designated tsA58 (iMHCK7.Tag-tsA58). The addition of doxycycline results in dedifferentiation and proliferation of the cells while its removal causes redifferentiation toward fully functional AMs. The 4th panel from the left describes the use of (i)PSCs, which are generated either by the disruption of embryos (ESCs) or by reprogramming somatic cells (iPSCs) using viral (e.g., Sendai virus) or non-viral vector-based methods. The (i)PSCs can proliferate virtually indefinitely and be differentiated in a mixed population of immature CM-like cells by a multistep procedure recapitulating the different stages of cardiac development and involving the use of specific inducers. After cell sorting/enrichment, these cells as well as the cells generated by the other techniques, can be used for different applications as depicted in the lower panel. The lower right picture merely serves to indicate that AMs/atrial tissue constructs might be applied in the future for reparative purposes (like the repair of atrial septal defects) and does not want to suggest their suitability for the regeneration of ventricular tissue.

In the next sections, the different 2D and 3D multicellular *in vitro* models that are used to study atrial arrhythmias will be described. Of each model, we will highlight its origin, mention key publications, advantages and limitations and discuss implications for future studies. For a brief summary, see Table 1. Also included is a schematic overview (Figure 1) of the various methods used to generate the models and of their (potential) applications in disease modeling, fundamental research, drug screening and regenerative medicine (e.g., the repair of atrial septal defects).

**Table 1:** Overview of the different 2D multicellular *in vitro* models of AF.

***In vitro* 2D model**	**Key publication**	**Origin**	**Advantages**	**Limitations**	**Derived CM population**	**EP characteristics**	**Reentrant circuits**
Permanently immortalized AMs	Dias et al. (31). / HL-1 / 2014	Enzymatic digest of right atrial tumor of HsNPPA.Tag transgenic mice	- Affordable - Scalable to demand - Well-characterized cells -Reduces laboratory animal use	- Non-human AP - Immature & heterogeneous phenotype - Lack of proliferation control -Spontaneous reentrant waves	High structural heterogeneity (Actn2 staining)	CV: 0.5–4 cm/s APD_90_: 400–550 ms Depends on HL-1 subclone	Spontaneous presence of reentrant circuit Activation frequency: 2.83 ± 1.43–3.02 ± 0.56 Hz Arrhythmia complexity: 0.43 ± 0.19–1.12 ± 0.14 reentrant circuits/cm^2^
Primary (animal)-derived AMs	Bingen et al. (30). / NRAMs / 2013	Enzymatic digest of neonatal rat atria	- Affordable - Scalable to demand - Easy to establish -Fully functional CMs	- Non-human AP - Ethical concerns -Mixed population of non-CMs and CMs	83% AMs (Actn2 staining; 100 % Myl7^+^) and 17% non-CMs	CV: 22.4 ± 1.9 cm/s APD_80_: 54.0 ± 2.8 ms	Activation frequency: 14.1 ± 7.4 Hz Arrhythmia complexity: 6.7 ± 7.7 reentrant circuits per culture
Conditionally immortalized AMs	Liu et al. (32) / iAM-1 / 2018	NRAMs conditionally immortalized with iMHCK7.Tag-tsA58	- Affordable - Scalable to demand - Control over proliferation - Reduces laboratory animal use - Functionally resembling NRAMs -Phenotypic homogeneity	- Non-human AP - Less suitable for personalized disease modeling	Low structural heterogeneity (Actn2 & Myl7 staining)	CV: 21.5 ± 1.0 cm/s APD_80_: 54.3 ± 1.6 ms	Activation frequency: 18.9 ± 1.3 Hz Arrhythmia complexity: 1.9 ± 0.6 reentrant circuits per culture
(induced) Pluripotent stem cells	Laksman et al. (33). / hESCs / 2017	hESC line HES3 (Nkx2-5^egfp/w^)	- Well-suited for - personalized disease modeling (hiPSCs) - Reduces laboratory animal use	- Time-consuming - Immature & heterogeneous phenotype - Costly -Scalability issues	85% CMs (87% atrial - like, 7% nodal-like, 7% ventricle-like AP)	CV: 5.4 ± 1.3 cm/s APD_90_: 206 ± 73 ms	Activation frequency: 312 ± 30 ms (i.e., ~3 Hz) Arrhythmia complexity: Not described

*For each model the cellular origin, advantages and limitations are described as well as the key publication describing its use as multicellular AF model. Additionally, data about the identity/composition of the derived CM populations as well as their electrophysiological (EP) and reentrant wave characteristics are provided. Actn2, sarcomeric α-actinin. Myl7, atrial isoform of regulatory myosin light chain 2. For an explanation of the other abbreviations see the added list*.

### Primary (Animal-Derived) AMs

Until now, freshly isolated CMs of animals are the most widely used cell source for the generation of 2D models to study cardiac arrhythmias. Primary CMs are derived from heart tissue of various animal species by enzymatic digestion, resulting in cell suspensions that can be used to generate synchronized 2D cardiac syncytia. Initially, (transgenic) animal-derived primary CMs were mainly used for single-cell patch clamp studies (34–41), due to technical issues related to the accurate measurement of membrane voltage changes in monolayer cultures. The use of CM monolayers in cardiac arrhythmia research strongly increased after the development of (i) chemical and later genetic voltage and Ca^2+^ probes and (ii) advanced imaging systems for optical voltage and Ca^2+^ imaging with high spatial and temporal resolution (42). This made it possible to non-invasively study electrical impulse propagation on a microscopic and macroscopic level (43–45). The concept of reentry originated from basic experiments in a jellyfish shortly after the beginning of the previous century, which has matured over recent decades and reentrant conduction has also been shown in explanted cardiac tissue (46–48). Nevertheless, Bub et al. were the first to show in 2D cultures of embryonic chick heart cells the presence of spontaneous reentrant waves by the use of optical Ca^2+^ imaging, which resembled results obtained *in silico* (49–54). Afterwards, monolayers derived from neonatal rat ventricular CMs (NRVMs) were established as a model that could be used to induce anatomical and functional reentry. High-resolution optical voltage mapping made it possible to study in detail the mechanisms involved in (i) the initiation of reentry and (ii) the subsequent termination of reentrant tachycardias by rapid pacing and electrical shocks. (44, 55–61) Currently, NVRMs are the most widely used model to study cardiac arrhythmias as evidenced by the large number of research papers, in which these cells are used (62–65). This is exemplified by studies with NRVMs that looked at the effects of ischemia/reperfusion injury, anisotropy, fibrosis, CM-(myo)fibroblasts coupling, hypertrophy as well as changes in the activity of ion channels and gap junctions on cardiac arrhythmias (66–78). Although arrhythmia mechanisms uncovered by investigation of ventricular CMs may also be relevant for understanding the initiation and maintenance of atrial arrhythmias, there are marked differences between ventricular and atrial CMs on a structural and electrophysiological level (79–82), making it crucial to use atrial CMs for the modeling atrial arrhythmias like AF.

Initially, the multicellular modeling of atrial arrhythmias was based on computer simulations due to the lack of proper *in vitro* models (83). This changed when animal-derived atrial tissue was separated and used for CM isolation, resulting in studies that could use patch clamp as well as voltage- and Ca^2+^-sensitive dyes to study the dynamics of specific ion currents in an atrial context (84–86). More recently, Bingen et al. were the first to employ neonatal rat atrial CMs (NRAMs) for the generation of 2D atrial monolayer cultures, which were subsequently used to study electrical propagation by optical voltage mapping (30). Rapid electrical point stimulation of these cultures induced AF-like functional reentry (rotor frequency: 14.1 ± 7.4 Hz, rotor complexity: 6.7 ± 7.7 phase singularities). The authors showed that the rotor frequency and complexity was correlated with AP duration (APD) and that specific inhibition of I_KACh−c_, an atrial, acetylcholine-activated inward-rectifying K^+^ current known to be constitutively active in perAF (87, 88), by tertiapin resulted in APD prolongation and a subsequent decrease in rotor frequency and complexity (30). Similar effects were observed by knockdown of the expression of the molecular determinants of I_KACh−c_, Kir3.1 and Kir3.4 (89), indicating a potential role of these ion channels in AF dynamics. In a follow-up study, the same research group used confluent NRAM monolayers to study how (inhibition of) I_KACh−c_ affects the success rate of electrical cardioversion of AF (90). These studies inspired the generation of mono- and multicellular *in silico* NRAM models (91). Despite the promising results obtained with these rat models as well as *in vivo* using canine and ovine models of tachypacing-induced (persistent) AF (92, 93), no improvements in AF burden were seen in pAF patients treated with a potent and selective inhibitor of I_KACh−c_ (94), illustrating that promising *in vitro* or *in vivo* results obtained in animal models do not always translate into effective treatments for humans. A different and novel approach used to study the mechanisms involved in the termination of atrial arrhythmias was the optogenetic modification of NRAM monolayers, resulting in the expression in NRAMs of ion channels whose activity can be controlled with high precision in time and space by light (95, 96). The high spatiotemporal control of electrical depolarization allowed Feola et al. to demonstrate that the successful and local targeting of atrial rotors requires a line of conduction block that spans from the rotor core region to at least one unexcitable boundary (97). A combined *in vitro* and *in silico* follow-up study showed that optogenetics could also be used to manipulate the dynamical properties of spiral waves, by forcing local, depolarizing photocurrent-induced transitions between functional (freely rotating spiral waves) and anatomical (anchored reentrant waves) reentry through which rotor complexity could be reduced and reentrant conduction could be terminated (98). The findings of these studies help to understand how ablation strategies could result in the termination of atrial arrhythmias and AF. Additionally, these studies could aid in the refinement of rotor-based ablation strategies, thereby improving ablation efficacy and limiting tissue damage caused by this technique.

As presented, primary (animal-derived) AMs are a suitable model to study especially the (basic) electrophysiological mechanisms involved in the initiation, perpetuation and termination of atrial arrhythmias. The resulting insights could potentially lead to the development of new or improved therapies for the benefit of AF patients. Several advantages of the primary AMs cultures are that they represent well-established atrial models (protocols and basic electrophysiological properties available), are relatively easy to implement in a laboratory (cell procurement requires few reagents and no specialized equipment) and can be scaled according to research demands. An interesting recent development is the generation of a 3D model from primary neonatal rat atrial and ventricular cells as presented by Krause et al. (99). The resulting engineered heart tissues (EHTs) provide similar degrees of standardization and reproducibility as 2D systems but more closely resemble the spatial features of atrial and ventricular tissue (100). Comparison of the atrial and ventricular neonatal rat EHTs revealed marked differences in gene expression, contractility, electrophysiology and drug responses (99) providing a strong argument for the use of atrium-specific models to study atrial disease. The authors also found atrial neonatal rat EHTs to exhibit a more mature phenotype than EHTs created with human pluripotent stem cell (PSC)-derived atrial-like CMs. A limitation of using AMs isolated from heart tissue for modeling of atrial arrhythmias is the large effort, time and proficiency it takes to produce cell cultures of high enough quality and purity for arrhythmia research. Additionally shortly after birth the large majority of CMs in the mammalian heart stop dividing. As a consequence, primary mammalian CMs isolated from post-neonatal hearts cannot be amplified *in vitro* (101, 102). Another disadvantage of using primary CMs of animals as models of human cardiac arrhythmias are the known differences in cardiac electrophysiology, mainly due to differences in the composition, properties and activity of cardiac ion channels, between humans and the animal species commonly used for arrhythmia research (13, 103, 104). This limits the translational value of these models. An additional drawback is that the use of laboratory animals is subject to an ongoing ethical debate and limited within strict boundaries enforced by animal welfare committees (105). CMs derived from human PSCs, as will be discussed in the next paragraph, could be a suitable alternative for cardiac arrhythmia modeling by resolving the ethical and translational issues associated with the use of animal CMs for arrhythmia research.

### (Human) PSC-Derived Atrial(-Like) CMs

As mentioned, human PSCs hold great promise as CM source to build *in vitro* models for studying cardiac arrhythmias, especially since they can proliferate virtually indefinitely and are capable of differentiating into various types of CMs (106). Initially, human embryonic stem cells (hESCs), originating from the inner cell mass of blastocysts, were used as source of CM-like cells (107). After aggregation and exposure to differentiation conditions, hESCs spontaneous form contracting embryonic bodies (EBs), containing (partially) differentiated cells of all three germ layers (i.e., endoderm, mesoderm and ectoderm) including small clusters of phenotypically heterogeneous CM-like cells (108–111). The low yield and phenotypic heterogeneity of EB-derived CM-like cells limited their use in cardiac disease modeling, which initiated the development of novel differentiation protocols consisting of monolayer cultures, inductive co-cultures as well as directed differentiation procedures (112, 113). The latter, mainly driven by knowledge acquired from embryonic and fetal cardio(myo)genesis, resulted in relatively high yields of CM-like cells (up to 50%) (113–117). Further improvements were made by the genetic engineering of different reporter lines (e.g., NKX2-5^eGFP/w^ hESCs), allowing selection of relatively pure populations of (i) committed cardiac progenitor cells for subsequent cardiomyogenic differentiation or (ii) CM-like cells differentiated from hESCs (118, 119). However, the widespread use of hESC-derived CMs is hampered by ethical and political controversies due to the necessity of disrupting a human embryo to obtain hESCs (120). Therefore, the possibility to reprogram adult (human) somatic cells into induced pluripotent stem cells (iPSCs) by the use of four factors (OCT4, SOX2 and either KLF4 and MYC or NANOG and LIN28), revolutionized the use of PSCs. (121–124) Human iPSCs (hiPSCs) showed a similar cardiomyogenic differentiation potential as hESCs. Using optimized cardiomyogenic differentiation conditions it is now possible to derive CMs with efficiencies of ~95% from various hiPSCs and hESCs lines. The resulting CMs are, however, in general, relatively immature (i.e., resembling heart muscle cells at an early fetal stage of cardiac development) (125, 126) and typically consist of a mixture of ventricular (~60%), nodal (~30%) and atrial (~10%) CM-like cells, displaying highly heterogeneous electrical properties (106, 127, 128). Accordingly, to end up with more or less homogeneous populations of PSC-derived CMs various different selections strategies have been developed (129), which include the use reporter lines to select subtype-specific CMs by fluorescence-activated cell sorting (130, 131). More information on the currently available protocols to generate PSC-derived CMs and criteria to distinguish the different subtypes of PSC-derived CMs can be found in some recent reviews (116, 127, 132–134). An alternative strategy to generate CMs from non-CMs is through the direct cardiomyogenic reprogramming of fibroblasts by (cardiomyogenic) transcription factors, miRNAs or small molecules (135, 136). However, thus far, the reprogramming efficiencies are low and the resulting so-called induced CM-like cells (iCMs) are rather immature and comprise a mixture of different CM subtypes, limiting their suitability for cardiac disease modeling. Improvements in the cardiomyogenic differentiation protocols of PSCs and the generation of patient-specific hiPSCs made it possible to use human PSC-derived CMs as platform for disease modeling and drug screenings. (137, 138) Initially, the generated CM-like cells, which mainly possessed a ventricular phenotype, were used for drug screenings as well as to model monogenic arrhythmogenic diseases like channelopathies (138–141). These studies mostly involved the use of single cells or small cell clusters to assess, electrophysiological parameters like AP morphology and duration, the properties of (individual) cardiac ion currents as well as the occurrence of early and delayed afterdepolarizations (EADs and DADs, respectively). (138) There were only a few research groups capable of producing PSC-derived CM-like cells, first mainly with a ventricular-like morphology, in sufficient numbers to generate multicellular CM sheets suitable for studying the initiation and dynamics of cardiac arrhythmias as well as their termination by drugs or electrical shocks (142–147).

The use of PSCs in the modeling of atrial arrhythmias like AF started with the discovery that properly timed retinoic acid (RA) signaling stimulates the directed differentiation of human PSCs toward the atrial cardiac lineage (148–150), resulting in CMs with mainly an AM-like AP including the presence of atrium-specific currents (e.g., I_Kur_ and I_KACh_) that could be targeted with selective ion channel modulators (151–153). Initially, these cells were used to study the hereditary components in lone AF and recently also to investigate the effects of drugs on AF susceptibility (154, 155). The study by Shafaattalab and colleagues showed the Bruton's tyrosine kinase inhibitor Ibrutinib to induce pro-arrhythmic changes (i.e., APD shortening and DAD formation) in atrial-like but not in ventricular-like hESC-derived myocytes, emphasizing again the importance of using subtype-specific CMs for disease modeling (155). Laksman et al. were the first to generate sufficient numbers of atrial-like CMs to produce multicellular sheets that could be used to study mechanisms involved in atrial arrhythmias (33). This was accomplished by EB-based directed differentiation of hESCs (116, 119) with the addition of RA during the mesoderm stage. Subsequent fluorescence-activated cell sorting resulted in CM populations consisting of cells with (~87%) atrial-like, (~7%) ventricular-like and (~7%) nodal-like APs. The generated multicellular sheets showed an AP morphology resembling that of human adult atrial tissue (APD_90_: 206±73 ms) (156). Electrical propagation in these sheets was slow (5.4±1.3 cm/s), in comparison to adult human atrial myocardium (60-75 cm/s in bulk tissue) (157) and the earlier described NRAM and NRVM models (20–25 cm/s), but similar to the aforementioned ventricular-like CM sheets (142–147). The authors attributed the slow AP propagation to the relatively positive resting membrane potential of the cells. This resulted in a reduced availability of cardiac fast Na^+^ channels and therefore a low maximum upstroke velocity and small AP amplitude as manifestations of the immaturity of the cells. Nevertheless, rapid pacing of the cultures induced a reentrant wave rotating around an unexcited core with a cycle length (CL) of 312 ± 30 ms mimicking the self-sustaining drivers observed in AF (158). In the same study, the effects of the antiarrhythmic drugs flecainide (class Ic) and dofetilide (class IIIa) on APD, conduction velocity (CV) as well as reentry characteristics (e.g., CL and curvature) were investigated, which yielded results consistent with those obtained in animal studies (159, 160). Only dofetilide was able to sporadically (1 out of 10 cultures) terminate reentry. Notwithstanding their different modes of action and observed pharmacological effects, both drugs resulted in an increased temporal excitability gap (= CV^*^[CL-APD_90_]), suggestive of a common mechanisms involved in reentrant wave destabilization and arrhythmia termination. Despite the limited success rate of reentrant wave termination, this study exemplifies how these models could be used to comprehend the essential electrophysiological mechanisms involved in drug-induced arrhythmia termination. This study further highlights the feasibility to use atrial-like human PSC-derived CMs as platform for the discovery and development of atrium-specific antiarrhythmic drugs with a lower risk of ventricular proarrhythmia than existing antiarrhythmic agents. Recently, Tanwar et al. showed that instead of RA also Gremlin 2 could be used to stimulate the differentiation of ESCs into atrial CM-like cells (161).

The veno-atrial junction in the PV antrum is a major source of ectopic arrhythmogenic foci, which is believed to be caused by the extension of myocardial tissue sleeves along the PVs on the adventitial side (22, 162). This anatomy could favor source-sink mismatch causing conduction delays, wavebreaks, functional reentry and AF (162). Nevertheless, as mentioned earlier, there's an ongoing debate about the role of focal ectopic drivers from the PVs during the initiation, maintenance and perpetuation of AF. Therefore there's still an urgent need for relevant PV models to study the role of these drivers in AF (23). Nakanishi et al. used PSCs for the modeling of the veno-atrial junction in the PV antrum (163). Cell sheets were generated by using RA during the monolayer-based directed cardiomyogenic differentiation of hiPSCs, resulting mainly in CMs with an atrial-like phenotype (99.6 ± 0.3% cardiac troponin T^+^ cells, 96.1 ± 1.0% MLC2a^+^ and MLC2v^−^ cells). These cells were mixed in different ratios (i.e., 10:0, 7:3, 3:7 and 0:10) with atrial fibroblasts of human adults and applied to a mold consisting of two silicone holes (12 mm Ø) connected with each other by a canal with the same width or a much smaller width (1–2 mm; narrow isthmus model), thereby mimicking PV myocardial tissue. In monolayers containing 100% atrial-like hiPSC-derived CMs, a narrowed isthmus resulted, at higher pacing frequencies (>5 Hz), in conduction delay distal from the isthmus as well as various types of conduction blocks (intermitted regional block, 2:1 block, omnidirectional block). In co-cultures with fibroblasts conduction delay and block occurred already at lower pacing frequencies. However, this did not result in the formation of reentrant waves. This is currently the only study using atrial-like CMs to generate a surrogate model of the veno-atrial junction in the PV antrum. While the authors speculate that paracrine factors released by the cardiac fibroblasts or heterocellular coupling between these cells and the hiPSC-derived AM-like cells may explain the observed effects, this was not further investigated in their study. Moreover, there are marked differences in the ionic currents between the CMs in the PVs and AMs / hiPSC-derived atrial-like CMs (164), thereby limiting the clinical relevance of the model. Thus, there remains a demand for more sophisticated human models that closely mimic the complex structural and electrophysiological properties of the veno-atrial junction in the PV antrum in order to enable researchers to study the exact role of focal ectopic drivers from the PVs during the initiation, maintenance and perpetuation of atrial arrhythmias.

Overall, the number of studies using human PSC-derived AMs to investigate the mechanisms involved in atrial arrhythmias is still limited (33, 163). This is remarkable with so many researchers working on the development of PSC-derived CMs as platform for drug screenings, disease modeling and personalized medicine. The limited use of PSCs as well-established model in AF research could partially be explained by the fact that the differentiation of PSCs toward atrial CM-like cells is a laborious, time-consuming and costly endeavor and therefore hard to scale up. Moreover, PSC-derived AMs phenotypically resemble fetal AMs. This structural and functional immaturity, limits the use of these cells in adult cardiac disease modeling (80, 125, 165). Therefore, in order to increase the clinical relevance and predictive value of human PSC-based cardiac arrhythmia models, improved cardiomyogenic differentiation protocols that result in more mature and less heterogeneous CM (sub)populations are required. However, as many cardiac disorders including AF are accompanied by (partial) dedifferentiation of CMs and the reactivation of (specific) fetal genes in these cells (166), development of representative arrhythmia models may not always require the use of fully matured PSC-derived CMs. Human PSCs also remain a highly attractive cells source for personalized medicine applications due to the ease with which their genomes can be modified using state-of-the art genome editing technologies (167–169) allowing (i) the introduction of disease-associated genetic variants in PSCs of a healthy individual and (ii) ‘genetic curing’ of patient-derived PSCs (154, 170, 171). Of the various approaches to improve PSC-based arrhythmia models, the engineering of 3D cardiac tissue constructs including heart-on-a-chip devices is of particular interest as this will allow assessment of cardiac electrical activity in a more realistic setting than can be provided by 2D CM monocultures (165, 172–174). This is nicely illustrated by a recent study of Goldfracht et al., who generated ring-shaped atrial and ventricular EHTs from hESCs showing heart chamber-specific differences in gene expression, electrophysiology, contractile properties and pharmacology (175). The authors demonstrated the utility of their atrial EHTs for therapeutic testing by investigating the effects of pharmacological (i.e., flecainide and vernakalant) and electrical cardioversions on the efficacy of arrhythmia termination and the maintenance of normal rhythm. Different drug responses between atrial and ventricular EHTs were also found by Cyganek et al. using hiPSCs as CM source (174). Another way to increase the usefulness of PSC-derived cardiac arrhythmia models is by the modification of PSCs with genetically encoded voltage or Ca^2+^ indicators to facilitate (high-throughput) drug screenings and to investigate different CM maturation strategies (146, 176–178). In this context, also gene-based organelle probes and fluorescent protein-tagged sarcomeric components will be highly useful as long as they do not interfere with the normal activities/functions of CMs. Similar tools could also be used in combination with the lines of immortalized CMs that will be discussed in the next section.

### Immortalized AMs

Cardiac disease modeling would greatly benefit from the ability to amplify (mature) CMs *in vitro*. Unfortunately, mammalian CMs typically lose proliferation capacity early after birth due to (permanent) cell cycle arrest. (101, 102, 179–181) The cell cycle is tightly regulated by transcription factors, cyclins and cyclin-dependent kinases (CDKs) as well as upstream regulators including the pocket proteins (i.e., pRb/p105, pRb1/p107 and pRb2/p130), CDK inhibitors of the CIP/KIP and INK4 families and the tumor suppressor protein p53 (182–184). The identification and ectopic expression of viral oncogenes made it possible to “immortalize” post-mitotic cells resulting in their cell cycle reentry and subsequent proliferation (185). Especially the simian virus 40 large T antigen (SV40 Tag), due to its ability to suppress the activity of pRb and p53, has been extensively used to generate lines of permanently immortalized parenchymal mammalian cells (e.g., hepatocytes, neurons, skeletal muscle cells and so forth) that could be used as platform for drug screenings and disease modeling (185–188).

The use of immortalized cell lines for the modeling of cardiac diseases started with the establishment of various transgenic mice, each with a different promoter (derived from the human NPPA, mouse PR1, rat Myh6 and mouse Nkx2-5 gene) driving the expression in CMs of SV40 Tag, which resulted in cardiac tumor formation in the atria and ventricles of embryonic and adult mice (189–192). Tumor nodules isolated from the different transgenic mice strains, yielded the transplantable right atrial tumor cell lines AT-1, AT-2 and MCM-1 as well as the 1H and ECL-2 cells (of unspecified anatomical origin) (191–195). Enzymatically released tumor cells could be cultured *in vitro* but showed signs of dedifferentiation for the 1H, ECL-2, AT-2 and MCM-1 cells (192, 196–200). AT-1 cells, on the other hand, retained some characteristics of adult AMs. However, these cells could not be serially passaged or thawed from a frozen stock, requiring the isolation of a subpopulation, called HL-1, that could be expanded indefinitely under specific culture conditions (201, 202). Proliferating HL-1 cultures contain cells at different stages of cardiomyogenic differentiation including cells with structural and functional properties of CMs (201, 203). Attempts to use other viral oncogenes for the immortalization of CMs were less successful, making SV40 Tag the most widely used tool for the generation of cardiac muscle cell lines (204).

Brundel and co-workers were the first to subject HL-1 cells to prolonged (24 h) electrical tachypacing, which resulted in contractile dysfunction and aspects of the electrical and structural remodeling observed in AF (205, 206). This led to a number of subsequent studies in which tachypaced HL-1 cells were used for the identification of specific proteins and regulatory pathways involved in AF-related atrial remodeling (207–212). These studies also showed that the addition of certain drugs (i.e., calpain inhibitors and endoplasmic reticulum stress inhibitors) could decrease the myocyte remodeling processes. The identification of these pathways and their specific inhibitors could aid in the development of drugs that prevent structural remodeling and disease progression. Hong and co-workers reported the spontaneous occurrence of reentrant conduction in confluent monolayers of HL-1 cells (213). Moreover, first Umapathy et al. and later Tsai et al. demonstrated in HL-1 monolayers the presence of complex fractionated electrograms (214, 215) similar to those recorded in patients with pAF and perAF, where they could be used to guide ablation strategies (216). Unfortunately, several studies including the multi-center STAR-AF II trial showed no additional benefit for complex fractionated electrogram-guided ablation on top of PV isolation in patients with perAF (217–219). HL-1 cultures have also been used to study the effect of certain drugs on the persistence as well as complexity of fibrillatory conduction (220, 221). The aforementioned publications highlight the utility of the HL-1 cell line as model for atrial arrhythmias and for unraveling the molecular pathways involved in these diseases. Despite their merits as atrial disease model, HL-1 cells have a number of important drawbacks. Due to the permanent expression of SV40 Tag (and therefore lack of control over proliferation), individual HL-1 cells reside in different phase of the cell cycle causing large structural and electrophysiological heterogeneity between them. This may also explain the low CV of electrical impulses in HL-1 monolayers (221). Moreover, different laboratories use distinct subclones of HL-1 cells as well as HL-1 cells with a different passage history complicating the interpretation of data and detracting from the robustness and reproducibility of the model (31, 203). Another disadvantage of the HL-1 model is the inability to control the initiation of arrhythmias, which limits its usefulness for studying the electrophysiological mechanisms involved in the initiation, perpetuation and termination of atrial arrhythmias. This limitation could partially be overcome by the use of more homogenous HL-1 subclones showing improved electrophysiological properties. For example, Houston et al. used the HL1-6 cell line, a subclone of HL-1 that propagates APs at a higher speed than the original cell line, in combination with optical voltage- and Ca^2+^-sensitive dyes to study the characteristics and modulators of reentrant circuits (222). However, the results from this study should be interpreted with caution since the HL1-6 cultures of Houston and colleagues still contained considerable inhomogeneities, which are a predisposing factor for cardiac arrhythmias (223).

As mentioned above, the lack of control over viral oncogene expression and therefore proliferation limits the utility of permanently immortalized CMs as disease models and in studies of cardiac regeneration. In line with these findings, inhibition of DNA synthesis by mitomycin C treatment enhanced the cardiomyogenic differentiation of permanently immortalized ventricular CMs (224). This indicates that CM proliferation and differentiation are competing processes, providing a rationale for the conditional immortalization of cells (225). This resulted in various alternative conditional immortalization strategies, in order to generate differentiation-competent cardiac muscle cell lines (224, 226–234). Nevertheless, none of the conditionally immortalized cell lines yielded functional CMs that were qualified for subsequent use as an atrial arrhythmia model. These disappointing results prompted Liu et al. to test an alternative method for the generation of conditionally immortalized lines of CMs, which gave rise to the so-called iAM-1 cell line. This monoclonal line of conditionally immortalized neonatal rat AMs displays high proliferation capacity and fully preserved cardiomyogenic differentiation ability (32). The iAM-1 cell line was generated by transducing primary cells isolated from 2-day old neonatal rat atria with a repressor-based lentiviral Tet-on system (235) in which the muscle-specific promoter MHCK7 drives the expression of a temperature-sensitive mutant of SV40 Tag designated tsA58. Exposure of the iAM-1 cells to doxycycline induced expression of SV40 Tag-tsA58, resulting in dedifferentiation (as evidenced by loss of excitability and disassembly of sarcomeres) and subsequent proliferation of the cells. Removal of doxycycline abolished SV40 Tag-tsA58 expression, resulting in growth arrest and spontaneous cardiomyogenic differentiation into excitable and contractile cells with well-organized sarcomeres. Confluent monolayers of iAM-1 cells at day 9 of differentiation showed an average CV of 21.5 ± 1.0 cm/s and an average APD_90_ of 88.7 ± 5.4 ms as determined by optical voltage mapping. Importantly, high-frequency electrical point stimulation of differentiated iAM-1 monolayers induced the formation of rotors with frequency of 18.9 ± 1.3 Hz. Moreover, treatment of differentiated iAM-1 monolayers with tertiapin, resulted in APD prolongation and the inability to induce reentry in the cell layers by electrical burst pacing. These findings underscore the potential of iAM-1 cells as a model to study atrial arrhythmias. Due to the ease with which iAM-1 cells can be transfected, they have been used to investigate the impact of non-coding variants in AF-associated genes on the expression of these genes (236).

Overall, (conditionally) immortalized AMs created by transgenesis or *in vitro* (onco)gene transfer are a valuable alternative to primary AMs and (i) PSC-derived AM-like cells as model to study mechanisms involved in atrial arrhythmias. This especially holds true for the conditionally immortalized iAM-1 cells, which are superior in terms of structural and electrophysiological maturation as compared to the permanently immortalized HL-1 cells and (i)PSC-derived atrial-like CMs. An additional advantage of the iAM-1 cell line is its monoclonal origin and therefore consistent behavior over multiple cell passages, which makes it a robust and reproducible atrial model. Other advantages of (conditionally) immortalized AMs are their ease-of-use and the relatively lows costs to produce them in large numbers. Differentiation-competent lines of CMs can also help to reduce the use of animals for cardiac research similar to PSC-derived CMs. A disadvantage of the different lines of immortalized CM is the presence in their genomes of a viral oncogene, which (after reactivation) might cause genome instability and malignant transformation. Although this may not directly hinder *in vitro* studies, it will be a major concern for *in vivo* applications of the cells (237, 238). Another disadvantage of conditionally immortalized CM is that genome editing to generate “patient-specific” disease models is more challenging than for PSC-derived CMs due to the different possible outcomes of such a genetic intervention. This requires clonal expansion of the genome-edited cells, subsequent identification of cells with the desired genome change(s) and separation of these cells from those that did not become genetically altered or underwent undesired genome alterations. Such clonal selection is easier done with PSCs than with (conditionally) immortalized CMs. An interesting future application of the iAM-1 cell line is in the engineering of (complex) 3D cardiac constructs closely resembling atrial tissue *in vivo*. To further expand the utility of the iAM-1 cells, they could be equipped with optogenetic sensors and/or effectors as has been done for primary CMs as well as for the HL-1 cell line (96, 239, 240). Finally, capitalizing on the successful generation of fully differentiation-competent lines of rat AMs by conditional immortalization, the same research group is currently applying their inducible lentiviral vector-based SV40 Tag expression system for the establishment of differentiation-competent lines of human AMs.

## Summary

Several methods have been used to develop cell culture systems to study AF. Primary animal-derived CMs are still the most widely used model to comprehend the mechanisms involved in the initiation, continuation and termination of atrial (and ventricular) arrhythmias. However, especially due to the differences in cardiac ion channels and AP characteristics between AMs of humans and animals, cultures of animal-derived AMs may not always be representative for what happens in human AF patients. Moreover, there is increasing social pressure to reduce the use of animals for biomedical research. Use of AMs derived from human PSCs should potentially yield more relevant AF models than can be obtained with animal cells and hiPSCs offer the possibility to generate patient-specific AMs, opening up the avenue for personalized disease modeling and drug screening. Unfortunately, until now the immaturity and heterogeneity of hiPSC-derived AMs as well as limits in cost-effectiveness and scalability hamper their broad application as 2D (and 3D) models to study the pathogenesis of arrhythmias in general and, in particular, of AF. Generation of sufficient cells to establish confluent monolayers for AF modeling is possible with immortalized AMs. However, AP spreading in confluent monolayers of HL-1 cells is very slow due to the phenotypic heterogeneity inherent to these permanently immortalized cells. This limitation was overcome by the generation of the iAM-1 cells, which, due to their doxycycline-controlled cardiomyogenic differentiation ability can be used to establish 2D cultures showing fast spreading of APs and fibrillatory activity after burst pacing. Still, each of the models described above has its own strengths and weaknesses. The use of EHTs will further improve the relevance of multicellular *in vitro* systems for modeling of ventricular and atrial arrhythmias. A general concern that accounts for all described atrial arrhythmia models is the difficulty to mimic perAF, especially since this entity is typically preceded by extensive electrical, metabolic and structural remodeling affecting AP generation and propagation, Ca^2+^ handling and contractility. Co-culturing AMs with cardiac (myo)fibroblasts as well as modification of the properties of ion channels and gap junctions and the introduction of aspects of aging and (nitro-)oxidative stress by genetic or chemical means could help to improve the clinical relevance of (per)AF models. Also, the currently available 2D multicellular *in vitro* AF models all focus on anatomical and functional reentry and have not been designed to study the role of high-frequency focal activity on the genesis and dynamics of AF. Future AF models should also include focal AF sources (i.e., the PVs) to cover the full range of AF-related electrophysiological disturbances. Accordingly, the best model for a particular application depends on the specific research question(s) to be answered.

The utility and/or relevance of 2D multicellular *in vitro* systems for AF research could be further increased by (i) invention of simple and cheap techniques of (local) perfusion, anisotropic cell alignment and biochemical, electrical and mechanical stimulation, (ii) endowing cells with genetically encoded biosensors of e.g., voltage, cytosolic Ca^2+^ concentration, cyclic nucleotide content and redox state, (iii) development of robust and inexpensive methods for (further) cell maturation, (iv) detailed characterization of the cellular epigenome, transcriptome, proteome, metabolome and signalome, and (iv) inclusion of other (non)-cardiac cell types besides AM in realistic patterns. Given their relative ease-of-use and cost-effectiveness, 2D multicellular *in vitro* systems are expected to maintain a prominent place in fundamental and applied AF research and will play an important role in the development of novel anti-AF drugs and the identification of new therapeutic targets for AF.

## Author Contributions

PG, ST, DP, and AV: Writing, reviewing and editing of manuscript.

### Conflict of Interest

The authors declare that the research was conducted in the absence of any commercial or financial relationships that could be construed as a potential conflict of interest.

## Abbreviations

AF: Atrial fibrillation
AM(s): Atrial myocyte(s)
pAF: Paroxysmal atrial fibrillation
perAF: Persistent atrial fibrillation
AP(D): Action potential (duration)
CM(s): Cardiomyocyte(s)
iCMs: Induced cardiomyocyte-like cells
CDK(s): Cyclin-dependent kinase(s)
CL: Cycle length
CV: Conduction velocity
DADs: Delayed afterdepolarizations
EADs: Early afterdepolarizations
EHTs: Engineered heart tissues
EBs: Embryonic bodies
(h)ESC(s): (Human) embryonic stem cell(s)
NRVM(s): Neonatal rat ventricular CM(s)
NRAM(s): Neonatal rat atrial CM(s)
(h)(i)PSC(s): (Human) (induced) pluripotent stem cell(s)
PV(s): Pulmonary vein(s)
2D / 3D: Two-dimensional / Three-dimensional
RA: Retinoic acid
SV40 Tag(-tsA58): Simian virus 40 large T antigen(-tsA58).
